# A magnetic resonance imaging based radiomics model to predict mitosis cycles in intracranial meningioma

**DOI:** 10.1038/s41598-023-28089-y

**Published:** 2023-01-18

**Authors:** Hermann Krähling, Manfred Musigmann, Burak Han Akkurt, Thomas Sartoretti, Elisabeth Sartoretti, Dylan J. H. A. Henssen, Walter Stummer, Walter Heindel, Benjamin Brokinkel, Manoj Mannil

**Affiliations:** 1 University Clinic for Radiology, University Hospital Muenster, Westfälische Wilhelms-University Muenster, Albert-Schweitzer-Campus 1, 48149 Muenster, Germany; 2 Faculty of Medicine, University of Zurich, Zurich, Switzerland; 3 Department of Medical Imaging, Radboud University Medical Center, Radboud University, 6500HB, Nijmegen, The Netherlands; 4 Department of Neurosurgery, University Hospital Muenster, Westfälische Wilhelms-University Muenster, Albert-Schweitzer-Campus 1, 48149 Muenster, Germany

**Keywords:** Cancer in the nervous system, Cancer, Mathematics and computing

## Abstract

The aim of this study was to develop a magnetic resonance imaging (MRI) based radiomics model to predict mitosis cycles in intracranial meningioma grading prior to surgery. Preoperative contrast-enhanced T1-weighted (T1CE) cerebral MRI data of 167 meningioma patients between 2015 and 2020 were obtained, preprocessed and segmented using the 3D Slicer software and the PyRadiomics plugin. In total 145 radiomics features of the T1CE MRI images were computed. The criterion on the basis of which the feature selection was made is whether the number of mitoses per 10 high power field (HPF) is greater than or equal to zero. Our analyses show that machine learning algorithms can be used to make accurate predictions about whether the number of mitoses per 10 HPF is greater than or equal to zero. We obtained our best model using Ridge regression for feature pre-selection, followed by stepwise logistic regression for final model construction. Using independent test data, this model resulted in an AUC (Area under the Curve) of 0.8523, an accuracy of 0.7941, a sensitivity of 0.8182, a specificity of 0.7500 and a Cohen’s Kappa of 0.5576. We analyzed the performance of this model as a function of the number of mitoses per 10 HPF. The model performs well for cases with zero mitoses as well as for cases with more than one mitosis per 10 HPF. The worst model performance (accuracy = 0.6250) is obtained for cases with one mitosis per 10 HPF. Our results show that MRI-based radiomics may be a promising approach to predict the mitosis cycles in intracranial meningioma prior to surgery. Specifically, our approach may offer a non-invasive means of detecting the early stages of a malignant process in meningiomas prior to the onset of clinical symptoms.

## Introduction

Meningiomas remain the most common primary tumor entity of the central nervous system and are classified as grade I (about 80%), grade II (about 15%) and grade III (about 5%) lesions^1,2^. While prognosis is generally good, recurrence rates vary and distinctly rise with the WHO grade^3^. Despite molecular alterations becoming increasingly important for the diagnosis and prediction of the prognosis^4^, tumor grading still largely bases on histological criteria. The number of mitoses per 10 microscopic high-power fields is a key criterion for both diagnosis and differentiation of atypical (grade II) and anaplastic (grade III) meningioma^5^. In the WHO 2016 classification, brain invasion, in addition to a mitotic count of 4 or more, is a histological criterion that alone may be sufficient for the diagnosis of atypical meningioma, WHO grade II. An atypical meningioma can also be diagnosed based on the additive criteria of 3 of the other 5 histological features: spontaneous necrosis, sheeting (loss of whorling or fascicle architecture), prominent nucleoli, high cellularity and small cells (tumour clusters with high nuclear : cytoplasmic ratios)^5^. Anaplastic meningioma show a mitotic count of 20 or more per 10 HPF or papillary or rhabdoid histology^6^.

Treatment of meningiomas includes maximum safely achievable resection and/or irradiation therapy depending on patient status, tumor size, type and location.

However, a considerable portion of incidental meningiomas display no or slow tumor growth and can therefore be managed with a watch-and-wait strategy with serial magnetic resonance imaging^7^. However, unexpected (malignant) tumor growth can still occur thus leading to morbidity and, in some cases, also to the inability to perform radiosurgical treatment.

Prediction of tumor growth and progression in incidental, treatment-naïve meningiomas remains difficult but could offer distinct clinical benefits by allocating select patients early to treatment in case of evidence of tumor growth or progression.

In recent years, the field of radiomics has gained traction by expanding the diagnostic value of MRI through quantitative image evaluation. Specifically, by considering both clinical and histopathological data as well as quantitative MRI imaging data one can gain unprecedented insights into the current disease state or the expected course of a patient's disease^8–13^. Concerning meningiomas, a few previous studies have explored the utility of radiomics for the prediction of recurrence in surgically treated meningiomas^14^. Previous studies have already shown that statements regarding the probability of complete surgical resectability of meningiomas can be made by radiomics-based analysis of MRI images^15^. Furthermore, there are research approaches that evaluate semantic features such as heterogeneity of the tumour tissue, external shape or the mass effect caused by the tumour using radiomics-based analysis for meningioma grading^16^. However, its applicability in predicting distinct histological features as required for tumor grading remains largely unexplored^17,18^.

Given the key role of the mitotic count for meningioma grading, we aimed at analyzing the utility of radiomics as applied on preoperative imaging to predict mitotic count in a series of 167 neuropathologically confirmed, intracranial meningiomas. Therefore, we trained and tested machine learning models to distinguish cases with zero mitoses per 10 HPF (high power field / main field of view of histopathological assessment under the microscope) from cases with more than zero mitoses using pre-treatment MR images and radiomics. Additionally, we analyzed the performance of our final model as a function of the actual number of mitoses per 10 HPF.

## Methods and material

### Patient population

We retrospectively studied a patient collective of 167 consecutive patients who underwent surgery for intracranial meningioma at the Department of Neurosurgery, Muenster University Hospital, between 2015 and 2020. The patient collective consists of 148 patients with an initial diagnosis of meningioma and 19 of whom had a recurrence. Diagnosis and grading were performed according to the 2016 WHO Classification of brain tumors in all cases^5^. Hence, mitotic count in the tumor tissue was neuropathologically evaluated on hematoxylin and eosin (HE)-stained microscopic slices in 10 representative high-power fields (magnification 10×). Other inclusion criteria were: availability of a preoperative MRI contrast-enhanced T1-weighted MRI images (details see below) and availability of mitotic count on neuropathological reports.

The study was approved by the local ethics committee (AZ: 2018-061-f-S). This study was performed in line with the principles of the Declaration of Helsinki.

### MRI imaging and data pre-processing

MRI examinations were performed at Muenster University Hospital as well as at external clinics on 1.5–3 T scanners of different manufacturers (Siemens Healthineers and Philips Healthcare) after administration of 0.1 ml/kg of a contrast agent containing gadolinium (Gadovist, Bayer). A total of 89 patients were examined on 1.5 T scanners, 78 patients on 3 T scanners, 122 patients were examined in external clinics, 45 at Muenster University Hospital.

The MRI images were normalized with respect to their image intensities before further analysis to ensure comparability between the MRI images of different scanners.

The MRI images were evaluated by a radiologist with at least 6 years of experience in neuroradiological diagnostics blinded to any clinical or histological data.

**Figure 1:**
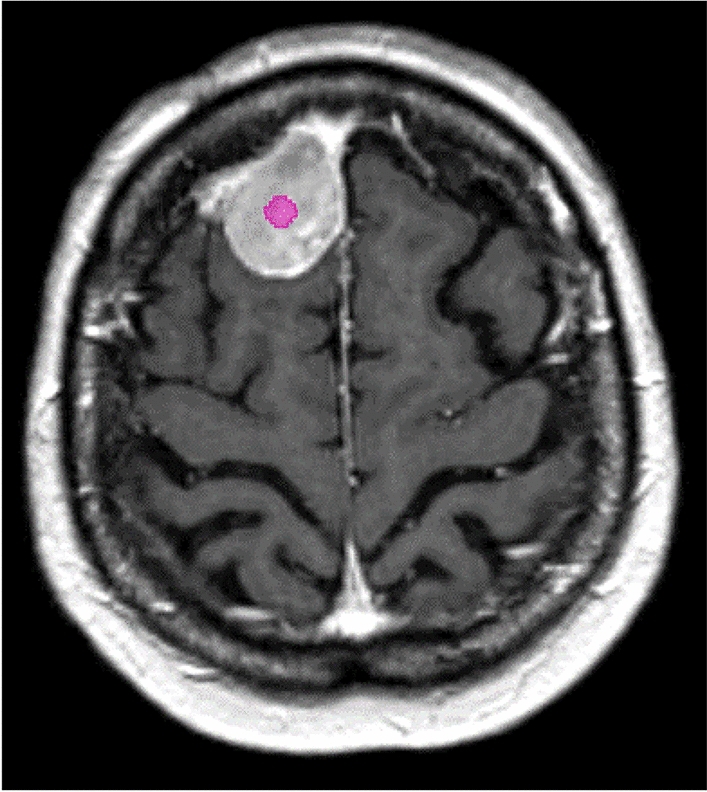
PyRadiomics segmentation: right high-frontal meningioma with an exemplary segmentation inserted as centrally as possible (pink circle, region of interest (ROI)). All segmentations had the same standardized size in each evaluation and were not allowed to contain necrotic or cystic tumor components.

Special care was taken that the regions of interest (ROI) were drawn with a standardized size as central as possible in the tumor mass and were delineated on a sequence of at least 5 slices (minimum slice thickness 1.5 mm). Additionally, the ROIs were not allowed to contain cystic or necrotic tumor components (Fig. 1).

In a further step, the acquired MRI images were processed in a standardized way using the 3D Slicer software and the PyRadiomics plugin (available at http://slicer.org/)^19^. PyRadiomics was used to obtain a total of 145 first-order statistics or features (variables), all based on the T1-weighted MRI images after contrast agent administration.

### Statistical analysis

Statistical analysis was performed using R software (version 3.5.3). Our final cohort of 167 patients included 107 cases with zero mitoses per 10 HPF and 60 cases with more than zero mitoses per 10 HPF. We divided the total of 167 data sets into a training and a test sample in a ratio of 4:1. The number of mitoses per 10 HPF (= 0/ > 0) and gender (female/male) were equally distributed in both samples (Table 1). The training data were used to construct the models and the independent test data to determine model performance with independent/unknown data.

**Table 1:** Histopathological and demographic distribution of the patient collective.

	Training data	Test data
Total number	133	34
Number of mitoses per 10 HPF (in%)		
= 0	63.91	64.71
> 0	36.09	35.39
WHO grade (in %)		
I	96.24	91.18
II	3.01	8.82
III	0.75	0.00
Gender (in %)		
Female	70.68	70.59
Male	29.32	29.41
Mean age (in years)	59.27	61.65

A two-step approach was used to construct the models. In the first step, the most important (most discriminatory) features were determined (feature preparation and pre-selection). In the second step, the number of features used was further reduced and the final models were estimated. Both steps were performed with the training data only.

In the first step, all features were z-score transformed and then subjected to a 95% correlation filter to account for redundancy between the features. Subsequently, the 20 most important variables were determined from all remaining variables using eight different algorithms for feature pre-selection. We used the R function "VarImp" to determine these most discriminant features. This function calculates the respective performance gain that results for each variable included in a model. In detail, the following eight algorithms were used for feature pre-selection:

1. Lasso regression
2. Ridge regression
3. Elastic-net regression
4. LDA (Linear Discriminant Analysis)
5. Distance correlation
6. Random forest
7. Bagged trees
8. Naive Bayes.

The tuning parameters (hyperparameters) included in these algorithms were determined using grid-search tenfold cross-validation (CV). Therefore, we divided the training data 10 times into groups with 90% and 10% of the training data, respectively. CV is a technique often used to achieve robust results with small data sets.

In the second step, the eight final models were created using stepwise logistic regressions. In each case, the 20 variables previously identified using the eight different feature pre-selection algorithms were used as the basis for the stepwise feature selection. The stepwise logistic regressions were performed using the "glmStepAIC" procedure in "R". This procedure selects the best combination of variables from a given set of variables and reduces the number of final model features as much as possible. Thus, the most discriminating multivariate feature combination is selected, which at the same time contains as few features as possible. Variables that contribute little or nothing to the discriminatory power of a model are removed from a model. To achieve this, our models were created using the Akaike information criterion (AIC). The AIC uses log-likelihood as a performance measure (maximum likelihood estimation). In addition, the AIC penalizes increasing model complexity depending on the number of parameters (variables) included in the model. Based on each of the groups of 20 variables we determined using the different feature pre-selection algorithms, we obtained final models including 6 (using random forest for feature pre-selection) to 16 features (using Ridge regression for feature pre-selection). The model optimization was performed for all models by maximizing of the Area Under the Curve (AUC) of the Receiver Operator Characteristic (ROC). We analyzed our models in terms of their ability to separate cases with zero or more than zero mitoses using AUC, accuracy, Cohen’s Kappa, sensitivity, specificity, positive predictive value and negative predictive value. In our analysis, sensitivity refers to the correct prediction of cases with zero mitoses and specificity to the correct prediction of cases with more than zero mitoses. The positive predictive value describes the proportion of correctly predicted cases with zero mitoses in relation to all predicted cases with zero mitoses. Accordingly, the negative predictive value describes the rate of correct predictions of cases with more than zero mitoses in relation to all predictions of cases with more than zero mitoses. In addition, we examined the performance of our final model as a function of the number of mitoses per 10 HPF.

### Ethics approval and consent to participate

This study was performed in line with the principles of the Declaration of Helsinki. Approval was granted by the Ethics Committee of the University of Muenster (Year 2018, No: 2018-061-f-S).

Informed consent was obtained from all individual participants included in the study.

### Human and animal ethics

All procedures performed in studies involving human participants were in accordance with the ethical standards of the institutional and/or national research committee and with the 1964 Helsinki Declaration and its later amendements or comparable ethical standards.

No procedures in this study were performed on animals.

## Results

### Model performance

Initially we aimed to distinguish cases with zero mitoses from those with more than zero mitoses. Table 2 summarizes the discriminatory power (AUC) obtained with the eight feature pre-selection algorithms followed by stepwise logistic regression for the training data and the test data respectively. With respect to the training data, all approaches resulted in models with AUCs of at least 0.77. The best model was obtained with the Ridge regression for feature pre-selection (AUC = 0.8912). Lasso regression, which is related to Ridge regression, achieved the best results on the independent test data. Overall, the AUC values obtained with the Lasso and the Ridge regression are very similar both on the training and test datasets respectively. A more detailed overview of the models’ performances on the independent test data is summarized in Table 3.

**Table 2:** Area under the curve (AUC) for the final models (training data and independent test data) using eight different feature-pre-selection algorithms and followed by stepwise logistic regression.

Feature pre-selection algorithm	AUC: training data	AUC: test data
Lasso regression	0.8838	0.8561
Ridge regression	0.8912	0.8523
Elastic net	0.8875	0.8220
LDA	0.8022	0.8371
Distance corr	0.8027	0.8447
Random forest	0.7730	0.8258
Bagged trees	0.8260	0.7538
Naive Bayes	0.8022	0.8371

**Table 3:** Classification results for the independent test data using eight different algorithms for feature pre-selection followed by stepwise logistic regression.

Performance measure	Feature pre-selection algorithm							
	Lasso regression	Ridge regression	Elastic net	LDA	Distance corr	Random forest	Bagged trees	Naive Bayes
Area under the curve (AUC):	0.8561	0.8523	0.8220	0.8371	0.8447	0.8258	0.7538	0.8371
Accuracy	0.8235	0.7941	0.7059	0.8235	0.8235	0.7941	0.7941	0.8235
Sensitivity	0.8636	0.8182	0.8182	0.9091	0.9091	0.8636	0.9091	0.9091
Specificity	0.7500	0.7500	0.5000	0.6667	0.6667	0.6667	0.5833	0.6667
Positive pred. value	0.8636	0.8571	0.7500	0.8333	0.8333	0.8261	0.8000	0.8333
Negative pred. value	0.7500	0.6923	0.6000	0.8000	0.8000	0.7273	0.7778	0.8000
Cohen’s Kappa	0.6136	0.5576	0.3307	0.5984	0.5984	0.5405	0.5221	0.5984
Number of final features	15	16	15	10	10	6	13	10

We determined our best model based on the training data, as only this data is known when developing a model. In our case, this was the model created with Ridge regression for feature pre-selection.

Using the independent test data, all eight approaches resulted in final models with an AUC of at least 0.754. However, with respect to all performance values listed in Table 3 (i.e. AUC, accuracy, sensitivity, specificity, positive predictive value, negative predictive value and Cohen's Kappa), the final models show different performances. This is especially true with respect to specificity. The best models have a specificity of 0.75, while the weakest models have a specificity of only 0.5. Among the eight models shown, only the Ridge regression and the Lasso regression resulted in final models that have a specificity greater than 0.7 both on the training data (not shown in the tables) and on the test data. The level of specificity achieved is obviously dependent on the number of final model variables used. This becomes even clearer when we examine the best model (using Ridge regression for feature pre-selection) in more detail.

### Model performance as a function of the number of the features included

Based on the model with 16 variables, we analyzed whether the number of model variables could be further reduced without compromising model performance.

**Figure 2:**
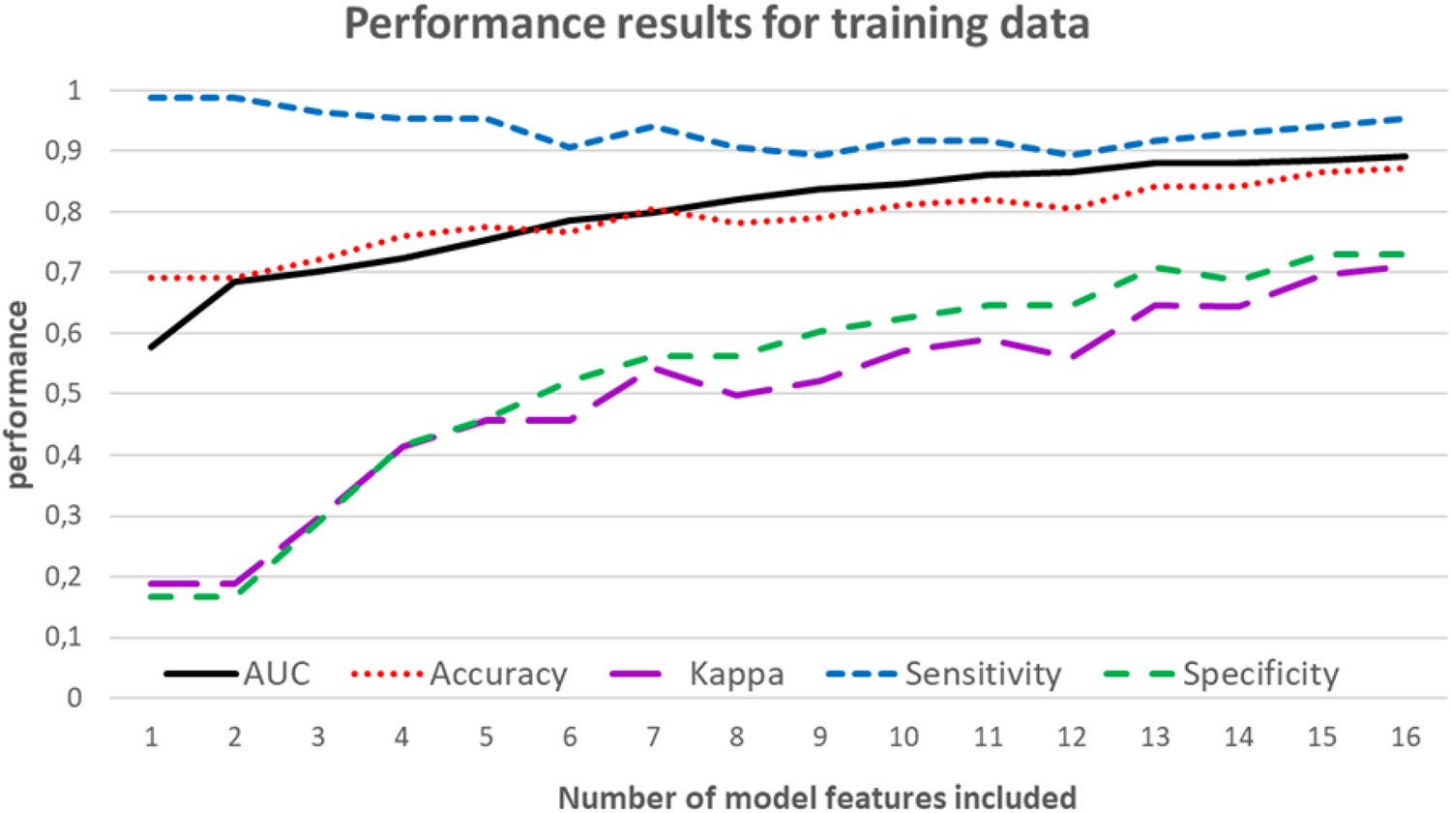
Development of the discriminatory power of the model (using Ridge regression for feature pre-selection) as a function of the number of features included. The performance values obtained with the complete model (including 16 features) are shown on the far right.

Figure 2 shows the development of the discriminatory power of the model with respect to the training data as a function of the number of features included when using Ridge regression for feature pre-selection followed by stepwise logistic regression. The performance values shown on the far left were determined for the one-feature model. This model contains only the most important feature. The stepwise logistic regression adds an additional variable to the model at each step. The performance values obtained with the complete model (including 16 features) are shown in Fig. 2 on the far right.

The 16 final features were implemented in the model by the stepwise logistic regression in the following order:

1. Sheeting
2. oedema_volume_1_0 (oedema volume > 0ccm or = 0ccm)
3. increased_cell_density
4. orig.shape.Sphericity
5. orig.gldm.DependenceVariance
6. orig.glszm.SizeZoneNonUniformity
7. orig.glcm.ClusterShade
8. Location_encoded_3 (tumor location = skull base: yes/no)
9. postop_volume
10. Age
11. orig.fst.ord.Skewness
12. KPI_g_80 (KPI greater than 80, i.e. KPI = 90 or KPI = 100: yes/no)
13. orig.glszm.GrayLevelNonUniformity
14. orig.glszm.LargeAreaLowGrayLevelEmphasis
15. orig.fst.ord.Kurtosis
16. Location_encoded_4 (tumor location = posterior fossa: yes/no).

As follows from Fig. 2, the discriminatory power increased sharply with the number of features used, especially with regard to specificity (and accordingly Cohen’s Kappa). Reducing the model to significantly less than 16 indicators is associated with a correspondingly large loss of discriminatory power. A significant reduction in the number of variables used is therefore not recommended.

**Figure 3:**
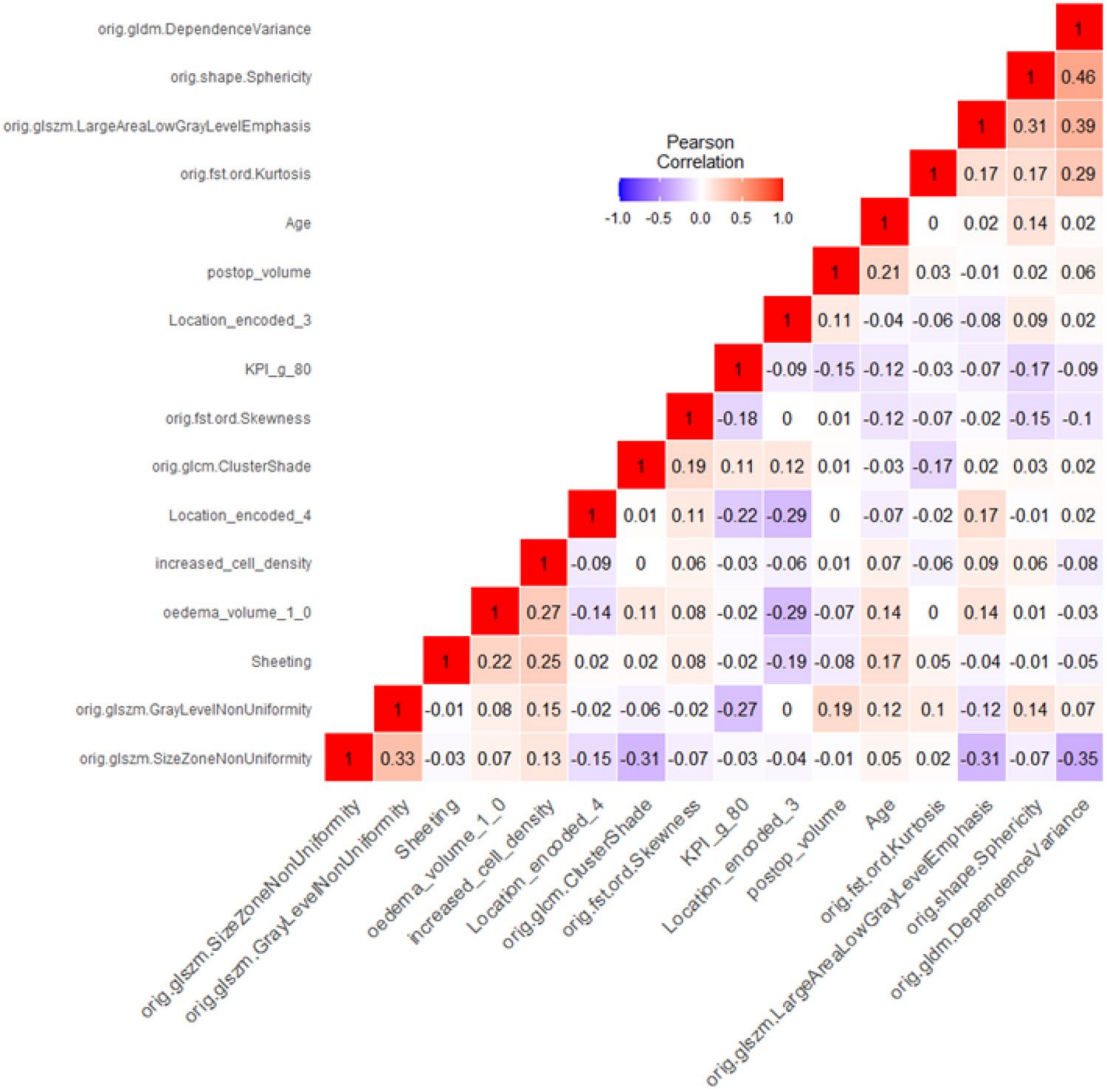
Pearson correlation matrix including 16 features.

**Figure 4:**
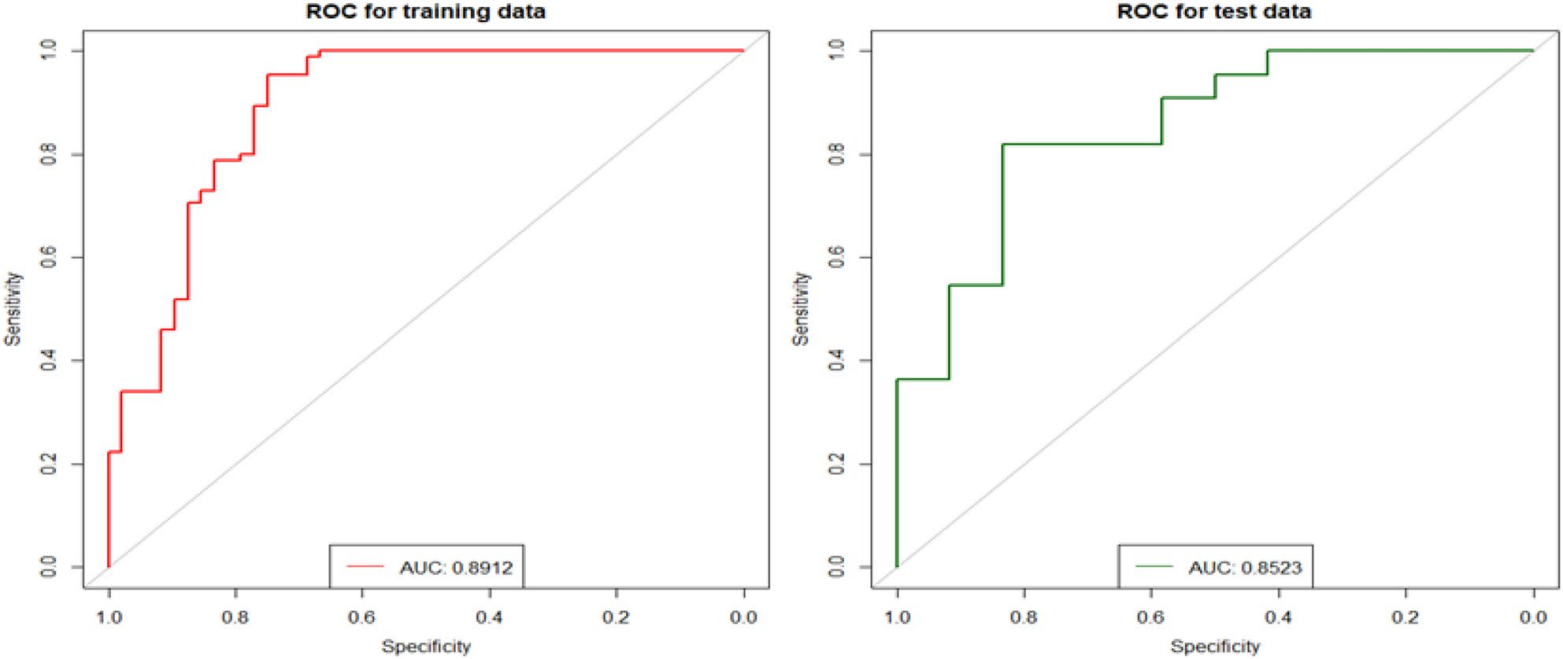
Receiver operating characteristics (ROC) for training and test data using Ridge regression for feature pre-selection followed by stepwise logistic regression. Final model includes 16 features. AUC: area under the curve.

The correlation matrix for this final model with 16 features is shown in Fig. 3. Most of the correlation coefficients are small thus signifying that most features are only slightly dependent on each other. In general, highly correlated variables should be avoided in multivariate models. This final model performed well on both the training data and the independent test data. The complete classification results, including the 95% confidence intervals for the training and test data are shown in Table 4. The receiver operator characteristics are shown in Fig. 4. It should be noted that we were able to obtain this model using two different approaches. First, with a forward procedure that started with zero variables, and second, with a backward procedure that started with all 20 variables. It should also be noted here that the choice of features included in the model and the performance of the model itself depend slightly on the used division of the data into training and test data. However, other data partitions we tested yielded similar models with comparable significant discriminatory power.

**Table 4:** Classification results for training and test data using Ridge regression for feature pre-selection followed by stepwise logistic regression. Values in brackets: 95% confidence interval.

Performance measure	Training data	Test data
Area under the curve (AUC):	0.8912 [0.8213: 0.9490]	0.8523 [0.6947: 0.9712]
Accuracy	0.8722 [0.8120: 0.9248]	0.7941 [0.6471: 0.9118]
Sensitivity	0.9529 [0.9041: 0.9890]	0.8182 [0.6429: 0.9565]
Specificity	0.7292 [0.6000: 0.8511]	0.7500 [0.4736: 1.0000]
Positive predictive value	0.8617 [0.7895: 0.9271]	0.8571 [0.6957: 1.0000]
Negative predictive value	0.8974 [0.7941: 0.9767]	0.6923 [0.4167: 0.9231]
Cohen’s Kappa	0.7111 [0.5776: 0.8302]	0.5576 [0.2429: 0.8211]

### Model performance as a function of the number of mitoses per 10 HPF

**Figure 5:**
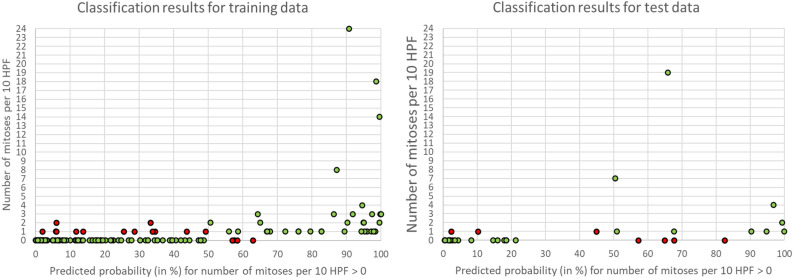
Classification results for the training and test data as a function of the probability that the number of mitoses per 10 HPF is greater than zero. Green dots represent correctly classified cases and red dots incorrectly classified cases.

Finally, we examined the performance of our final model (model using Ridge regression for feature pre-selection) in discriminating between cases with zero and more than zero mitoses per 10 HPF as a function of the actual number of mitoses. Figure 5 shows the classification results for the training and test data as a function of the probability that the number of mitoses per 10 HPF is greater than zero, and additionally as a function of the number of mitoses per 10 HPF. Green dots represent correctly classified cases and red dots incorrectly classified cases.

In a perfect model, all cases with zero mitoses would be placed in the lower left corner (at 0% and 0 mitoses) and all cases with more than zero mitoses would be placed on the right side (at 100% and more than 0 mitoses).

The figures show that most prediction errors occur in the case of one mitosis per 10 HPF. Table 5 summarizes the proportions of correctly predicted cases for 0, 1, 2, 3, 4, 5 to 10 and more than 10 mitoses per 10 HPF. In brief, cases with 0 mitoses per 10 HPF are predicted accurately while cases with 3 or more mitoses per 10 HPF are predicted even more accurately. Specifically, for the latter there are no prediction errors neither in the training nor in the test data. However, it must be clearly pointed out that our database contained only a few cases with 3 or more mitoses per 10 HPF. We therefore do not exclude that the actual prediction error rate for these cases could also be somewhat higher. We tried to distinguish cases with zero mitoses from cases with more than zero mitoses. Therefore, it is not surprising that most errors occurred in the transition region of our model, i.e. for the cases with one or two mitoses per 10 HPF.

**Table 5:** Proportions of correctly predicted cases of the training and test data from 1 to 10 mitoses per HPF (high power field/main field of view of histopathological evaluation).

Number of mitoses per 10 HPF	Training data		Test data	
	Total number of cases	Correctly predicted cases (%)	Total number of cases	Correctly predicted cases (%)
0	85	95.3	22	81.8
1	29	62.1	8	62.5
2	8	75.0	1	100.0
3	6	100.0	0	NA
4	1	100.0	1	100.0
5 to 10	1	100.0	1	100.0
> 10	3	100.0	1	100.0
Total/mean	133	87.2	34	79.4

## Discussion

Extent of resection or WHO grade can be used for estimating the risk of recurrence in surgically treated meningiomas^3,20^, however, prediction of progression or/ and high-grade histology in treatment-naïve tumors remains a key challenge during neuro-oncological care. Identification of risk factors for progression could greatly impact treatment recommendations and alter decision-making towards surgical or radiation therapy. A previous systematic review showed that individual analyses of imaging characteristics on preoperative MRI by radiologists do not allow for a reliable prediction of either high-grade histology or progression^21^. However, this may be partially caused by high interobserver-variability and subjectivity inherently associated with human assessments. In contrast, radiomics-based imaging analyses provides both objective and reliable data as well as high-through-put analyses for large patient series. Over the last years, a number of studies have shown that radiomics-based prediction of high-grade histology and sufficient assessment of the risk of postoperative tumor progression is feasible^12,14,22^. However, only few studies have focused on radiomics-based prediction of distinct prognostic histological characteristics. Exemplarily, some studies have shown the value of radiomics for the prediction of brain invasion, a stand-alone grading criterion for atypia since the WHO Classification of Central Nervous System Tumours in 2016^5,18,23–25^.

The upregulation of cellular growth factor signaling pathways, which results in the increase of tumor mass is always characterized by subsequent increased mitotic cycles of tumor cells^26–28^. In meningiomas, increased proliferation in terms of the Ki67 labeling index (LI) or the number of mitoses have been identified as strong predictors for recurrence, whereby the number of mitoses per 10 HPF directly impacts meningioma grading. Notably, Khanna et al. showed prediction of the Ki67 (LI) in grade I meningiomas using radiomics with very good sensitivity and specificity^17^. The advantage of a reliable prediction of the current mitotic number of a meningioma seems to us to be superior to other approaches to meningioma grading, as the histological grading of menigiomas defined in the WHO classification can be used directly.

To the best of our knowledge radiomics-based analyses of the mitotic count have not been investigated to date.

Our study shows that radiomics-based machine learning is a promising approach to detect the presence of current mitosis in intracranial meningiomas. Additionally, our study demonstrated that an accurate differentiation of the mitotic rate of meningiomas is possible and that a mitotic rate of more than 4 mitoses per 10 HPF, which is highly indicative of a malignant process, can be selected very accurately by our model. Increased mitotic count reflects biologically aggressive behavior. Hence, our approach may allow the identification of meningiomas at risk for progression, which then impacts preoperative decision-making. Notably, we believe that this approach may also allow physicians to reduce the number of pre-operative biopsies for neuropathological analyses if a comparable level of sensitivity and specificity can be achieved through image analyses.

This study has limitations that must be addressed. Specifically, this was a retrospective study. Moreover, imaging had been performed on various MRI scanners with different magnetic field strengths. The determination of the size of the examination volume to a defined and always the same measure has the consequence that tumour parts that are histologically possibly even more degenerated than the examination volume are not recorded. However, this problem also exists with every surgical biopsy; here, too, only part of the tumour can be histologically confirmed. As already noted, our database contained comparatively few cases with more than one mitosis per 10 HPF. However, the high sensitivity and specificity for the prediction of mitoses in our series reflect robustness of our approach as well as its broad applicability. Future analyses should include larger number of patients and cases with high-grade tumors as well as analyses of imaging performed under standardized conditions.

## Conclusion

In conclusion, our study demonstrates the potential of MRI-based radiomics models in detecting increasing cell mitosis as a reliable predictive marker of expected malignancy of intracranial meningiomas. Radiomics-based image analyses of preoperative imaging could thus impact decision-making during neuro-oncological care of meningioma patients.

## Data Availability

The datasets generated during and/or analysed during the current study are available from the corresponding author on reasonable request.

## Abbreviations

MRI: Magnetic resonance imaging
HPF: High power field
AUC: Area under the curve
ROI: Region of interest
LDA: Linear discriminant analysis
CV: Cross validation
ROC: Receiver operator characteristic
LI: Labeling index
